# Eye fluke infection changes diet composition in juvenile European perch (*Perca fluviatilis*)

**DOI:** 10.1038/s41598-021-81568-y

**Published:** 2021-02-09

**Authors:** Jenny C. Vivas Muñoz, Christian K. Feld, Sabine Hilt, Alessandro Manfrin, Milen Nachev, Daniel Köster, Maik A. Jochmann, Torsten C. Schmidt, Bernd Sures, Andrea Ziková, Klaus Knopf

**Affiliations:** 1 Leibniz-Institute of Freshwater Ecology and Inland Fisheries, Müggelseedamm 310, 12589 Berlin, Germany; 2 Faculty of Life Sciences, Humboldt University, Invalidenstrasse 42, 10115 Berlin, Germany; 3 Aquatic Ecology, University of Duisburg-Essen, Universitätsstr. 5, 45141 Essen, Germany; 4 Centre for Water and Environmental Research, University of Duisburg-Essen, Universitätsstr. 5, 45141 Essen, Germany; 5 Environmental Campus Birkenfeld, University of Applied Sciences Trier, Post Box 1380, 55761 Birkenfeld, Germany; 6 Institute for Environmental Sciences, University of Koblenz-Landau, Fortstrasse 7, 76829 Landau/Pfalz, Germany; 7 Instrumental Analytical Chemistry, University of Duisburg-Essen, Universitätsstr. 5, 45141 Essen, Germany

**Keywords:** Freshwater ecology, Ecology, Stable isotope analysis

## Abstract

Intraspecific diet specialization, usually driven by resource availability, competition and predation, is common in natural populations. However, the role of parasites on diet specialization of their hosts has rarely been studied. Eye flukes can impair vision ability of their hosts and have been associated with alterations of fish feeding behavior. Here it was assessed whether European perch (*Perca fluviatilis*) alter their diet composition as a consequence of infection with eye flukes. Young-of-the-year (YOY) perch from temperate Lake Müggelsee (Berlin, Germany) were sampled in two years, eye flukes counted and fish diet was evaluated using both stomach content and stable isotope analyses. Perch diet was dominated by zooplankton and benthic macroinvertebrates. Both methods indicated that with increasing eye fluke infection intensity fish had a more selective diet, feeding mainly on the benthic macroinvertebrate *Dikerogammarus villosus*, while less intensively infected fish appeared to be generalist feeders showing no preference for any particular prey type. Our results show that infection with eye flukes can indirectly affect interaction of the host with lower trophic levels by altering the diet composition and highlight the underestimated role of parasites in food web studies.

## Introduction

There is broad consensus that parasites commonly contribute positively to biodiversity and that they can play important roles in structuring communities^1–4^. Currently there is mounting evidence that parasites influence the interaction strength between the host and other species, having important effects on the functional role of hosts in the ecosystem and the structure of food webs^1,3,5–10^. Parasites may influence energy transfer through the ecosystem via trophic cascades by inducing alterations on consumer-resource interactions^3^. Numerous studies have associated parasitic infection with changes in a wide range of host behaviour (e.g.^11–13^). One of the most widespread examples occurs in trophically transmitted parasites, when parasites alter their hosts’ behaviour or phenotypic traits to increase susceptibility to predation by the target host^13,14^.

By infecting a sensory organ such as the eyes, diplostomid trematodes can potentially impair their second intermediate host’s visual performance^15,16^. Diplostomid trematodes have a typical three-host life cycle. In general, eggs are produced by adult worms in the definitive host (typically a fish-eating bird) and released into the aquatic environment. After hatching from eggs the miracidia penetrate a suitable mollusc first intermediate host, where they multiply asexually to produce cercariae. The cercariae are released into the environment to seek a second intermediate host, in which the metacercariae develop. Finally, metacercariae together with their second intermediate host must be ingested by an appropriate definitive host to complete the life cycle^17,18^.

Among Diplostomidae*, Diplostomum* spp. and *Tylodelphys* spp. infect a wide range of fish species as second intermediate host targeting different parts of the eye, such as the lens, vitreous humour and retina. The internal structure of the eye represents an immune privileged structure^19^ and thereby eye flukes can escape the host immune defence. Behavioural studies have shown that the lens infecting *Diplostomum spathaceum* and the vitreous humour dwelling *Tylodelphys clavata* have important consequences on the detection of prey, predators and conspecifics (e.g.^20–23^). Our previous studies^23,24^ showed that *T. clavata* impaired foraging efficiency and competitive ability of European perch (*Perca fluviatilis*). More heavily infected fish consumed less of the available food than their less infected conspecifics. It is conceivable, that in a visual predator such as European perch^25,26^ eye fluke infection may result in a modified diet composition due to impaired prey detection ability, potentially a different foraging behavior and the need to compensate for reduced foraging efficiency and lower competitive ability caused by the infection with eye flukes.

European perch is a widely distributed fish species in the Palaearctic region and one of the most abundant fish species in northern-temperate lakes^27^. Additionally, it serves as intermediate host to a diverse number of eye fluke species. Throughout Europe, eye fluke infections are highly prevalent in perch populations and commonly multiple species have been recorded within a single host^28–34^. Thus, knowledge about parasite-induced changes in the diet of perch could contribute to the understanding of the role of eye flukes in lake food webs.

In perch populations intraspecific diet specialization has been associated with habitat and resources use. In general, it has been described that individuals specialize in feeding on either littoral or pelagic prey types. This specialization has been related to morphological intraspecific variation in favour of better utilization of different habitats and/or diets (resource polymorphism). In lakes, the littoral juvenile perch that feed mainly on macroinvertebrates have deeper bodies than the pelagic ones that feed on zooplankton^35–38^. However, within-habitat individual diet specialization has also been observed among juvenile perch, especially in the littoral zone, which is assumed to reduce intraspecific competition^39–41^. There is evidence that prey selection and the degree of individual diet specialization of juvenile perch is influenced by resource availability, interspecific competition and predation pressure^42–49^. Yet, the effect of eye fluke infection on diet composition has not been evaluated.

The aim of the present study was to test whether perch alter their diet composition as a compensatory mechanism for impaired visual performance caused by eye fluke infection. Young-of-the-year (YOY) perch were sampled from Lake Müggelsee (Berlin, Germany) in two years, their eye flukes were counted and their diet was evaluated using stomach content analysis (SCA) and stable isotope analysis (SIA) of carbon (δ^13^C) and nitrogen (δ^15^N). Juvenile perch of this lake were known for a high variability in eye fluke infection intensity^34^. Additionally, in littoral habitats with abundant reed it has been observed that some individuals foraged exclusively on zooplankton while others had mixed diets with macroinvertebrates and zooplankton^50^. We hypothesized that YOY perch individual diet composition is influenced by eye fluke infection intensity with a higher share of benthic macroinvertebrates in the diet of fish with higher infection intensities. Consequently, eye fluke infections would modulate predation pressure on different prey organisms, and thus the functional role of perch in food webs.

Using two distinct techniques, SCA and SIA, enables to study the trophic ecology of perch over different timescales. SCA gives a short-term dietary “snapshot” of recently ingested items^51^. On the other hand, SIA provides temporally integrated information on dietary habits, reflecting what was actually assimilated by the consumer^52^. Differences in nitrogen and carbon isotope ratios reflect variance in diet and can reveal intra-population differences in diet preferences and give an indication whether an omnivorous population actually consists of generalist feeders or specialists with different preferences^53–55^. To estimate the contribution of specific prey items to fish diets, isotope data can be used in mixing models^56–58^. However, for these models, prior knowledge of the potential prey is necessary; therefore, the combined use of SCA and SIA provides a robust analysis in the evaluation of intra-population differences in diet composition.

## Results

### Eye fluke component community

YOY perch from Lake Müggelsee were infected with six species of eye flukes (see Table 1): *T. clavata*, *Tylodelphys podicipina*, *Diplostomum baeri* (sensu lato), *D. spathaceum*, an unidentified *Diplostomum* sp. and *Posthodiplostomum brevicaudatum*. The eye fluke component community was clearly dominated by *T. clavata* that represented 99.2% (2014 South: 96.4%, 2014 North: 99.3%, 2016 North: 99.5%) of all eye flukes. All sampled fish were infected with *T. clavata*, with the exception of one fish in 2016. The occurrence of the other eye fluke species was considerably lower; the second most common species was *D. baeri* (sensu lato) with a prevalence of up to 41.3%. A single perch was infected with *T. podicipina* (Table 1). Besides the clear dominance of *T. clavata*, eye fluke infection intensity varied greatly between the three sampling dates. In 2014, infection intensity in fish at the northern shore was seven times higher than in fish at the southern shore. Also the highest number of metacercariae recorded in a single fish was much higher for the northern shore (298) than for the southern shore (< 50). In 2016 eye fluke infection intensity at the northern shore was half of that recorded in 2014 at the same location (Table 1).

**Table 1:** Prevalence and mean intensity ± S.D. of trematodes infecting the eyes of YOY perch (*Perca fluviatilis*) from two sampling sites (“South” and “North”) of Lake Müggelsee in 2014 and 2016.

Species	Prevalence (%)			Infection intensity (metacercariae per fish)		
	South 2014	North 2014	North 2016	South 2014	North 2014	North 2016
*Tylodelphys clavata*	100	100	99.2	12.8 ± 9.5	92.1 ± 55.5	40.1 ± 19.2
*T. podicipina*			0.8			1.0 ± 0.0
*Diplostomum baeri* (sensu lato)	17.8	41.3	4.2	1.3 ± 0.6	1.6 ± 1.1	2.0 ± 1.7
*D. spathaceum*	1.1		0.8	1.0 ± 0.0		1.0 ± 0.0
*Diplostomum* sp.	2.2	0.4	4.2	1.0 ± 0.0	1.0 ± 0.0	1.4 ± 0.5
*Posthodiplostomum brevicaudatum*	13.3	2.7	3.4	1.7 ± 1.2	1.1 ± 0.4	1.5 ± 0.6
All species	100	100	99.2	13.3 ± 9.6	92.8 ± 55.7	41.0 ± 19.4

**Figure 1:**
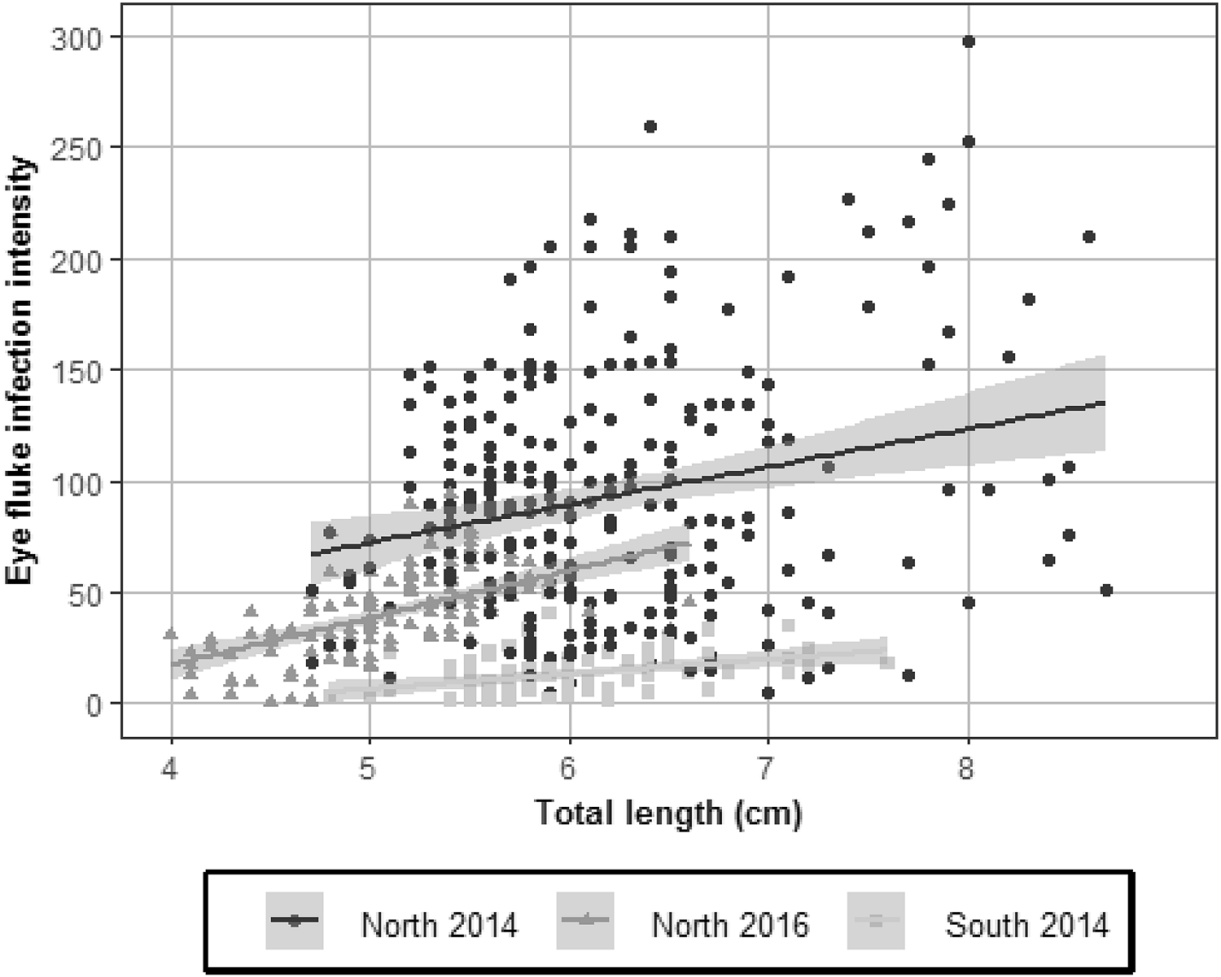
Relationship between eye fluke infection intensity and the total length of YOY perch (*Perca fluviatilis*) from two sampling sites of Lake Müggelsee (“South” and “North”) in 2014 and 2016. The lines represent the best linear fit (South 2014: *y* = 6.66*x* – 26.86; North 2014: *y* = 16.94*x* – 12.22; North 2016: *y* = 21.23*x* – 67.4957). The grey shading represents 95% confidence intervals.

Eye fluke infection intensity was positively related to body size of YOY perch at all sampling dates (South 2014: R^2^ = 0.13, *F*_1,88_ = 13.73, *P* < 0.001; North 2014: R^2^ = 0.06, *F*_1,257_ = 16.03, *P* < 0.0001; North 2016: R^2^ = 0.32, *F*_1,117_ = 55.95, *P* < 0.0001; Fig. 1). The size-dependent infection intensity among the two sampled years in the northern shore differed in magnitude but maintained a relatively similar slope (Fig. 1).

**Figure 2:**
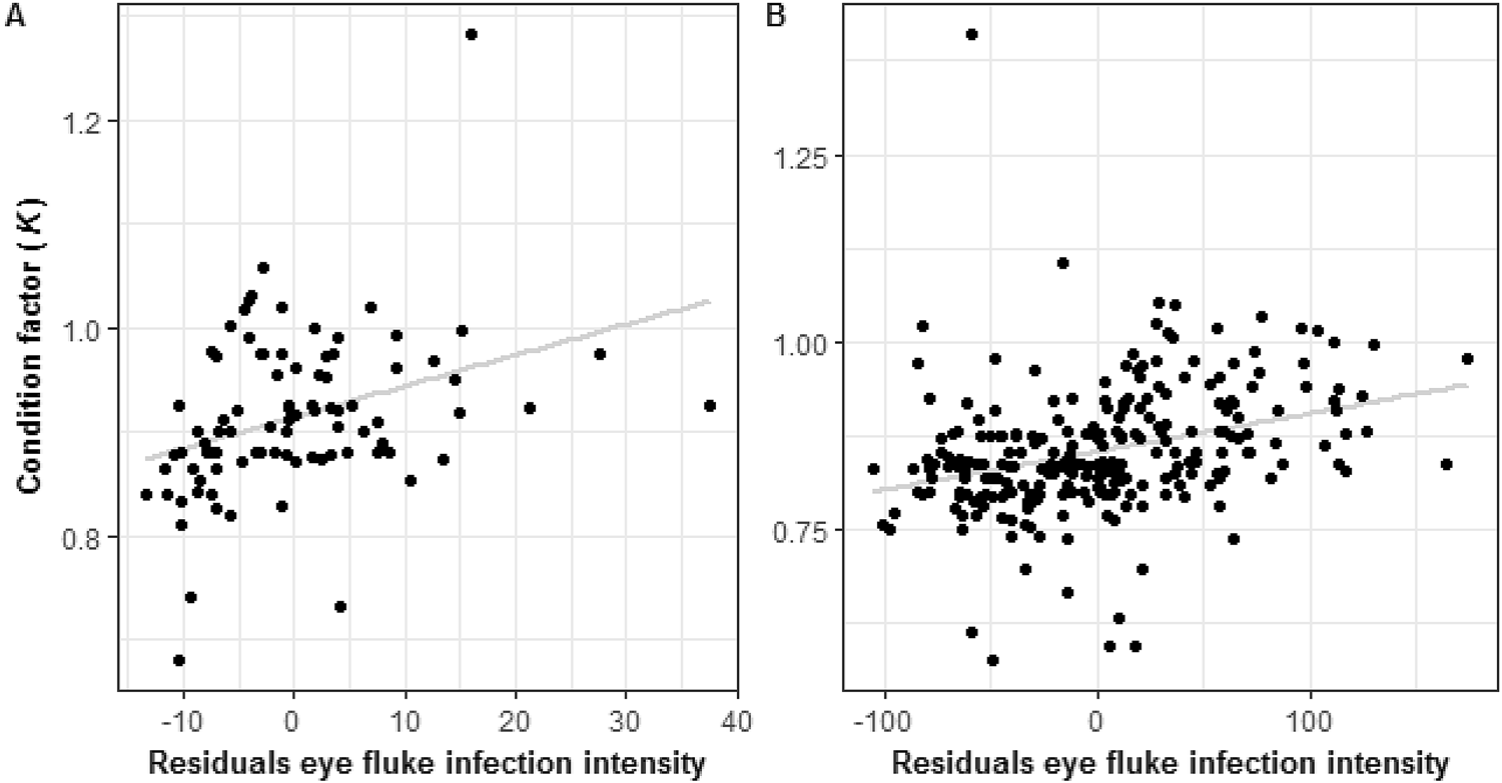
Relationship between eye fluke infection intensity corrected for fish size (residuals from the regression in Fig. 1) and condition factor (*K*) of YOY perch (*Perca fluviatilis*) from two locations of Lake Müggelsee (**A**) “South”, (**B**) “North” in 2014. The lines represents the best linear fit (South: *y* = 0.0029*x* + 0.874; North: *y* = – 0.00059*x* + 0.799).

Notably, a positive relationship between infection intensity (corrected for fish size) and *K* was detected for the two sampling sites in 2014 (Spearman’s rank correlation, South: *r*_*s*_ = 0.402, *n* = 90, *P* < 0.0001; North: *r*_*s*_ = 0.398, *n* = 259, *P* < 0.0001; Fig. 2A,B). In 2016, however, no significant correlation between infection intensity (corrected for fish size) and *K* was evident (*P* > 0.05).

### Stomach content analysis

**Figure 3:**
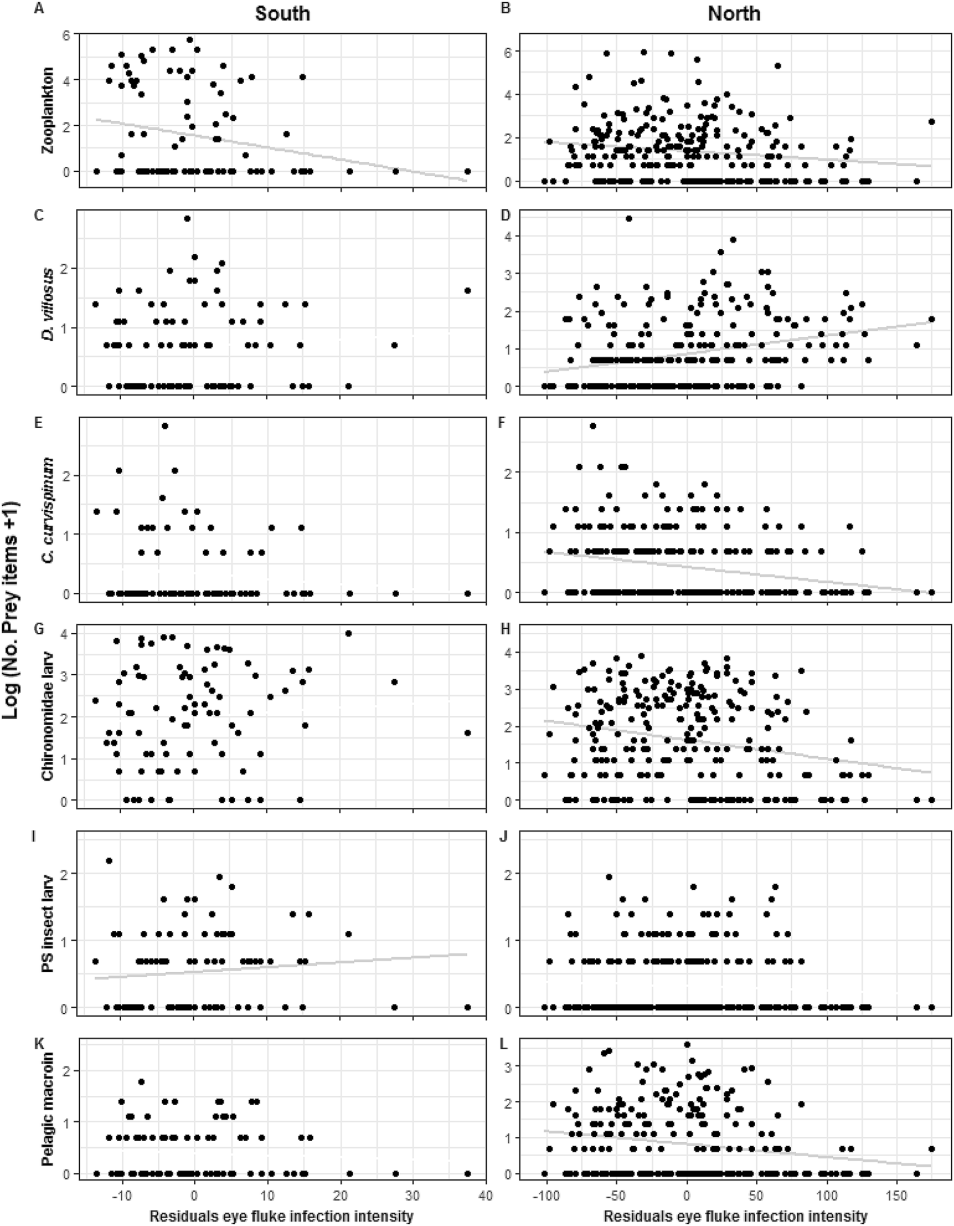
Eye fluke infection intensity corrected for fish size (residuals from the regression in Fig. 1) from perch (*Perca fluviatilis*) caught in two sampling sites of Lake Müggelsee in 2014 (left side: “South”, right side: “North”) and the number of consumed (**A**,**B**) zooplankton, (**C**,**D**) *Dikerogammarus villosus*, (**E**,**F**) *Chelicorophium curvispinum*, (**G**,**H**) Chironomidae larvae, (**I**,**J**) Predator-sensitive (PS) insect larvae and (**K**,**L**) pelagic macroinvertebrates. All prey data were log x + 1 transformed. Lines represent the best linear fit ((A) *y* = – 0.0052*x* + 1.551; (B) *y* = – 0.0041*x* + 1.363; (D) *y* = 0.0047*x* + 0.864; (F) *y* = – 0.0024*x* + 0.418; (H) *y* = – 0.0052*x* + 1.628; (I) *y* = 0.0071*x* + 0.524 (L) *y* = – 0.0037*x* + 0.813).

Stomach content analysis indicated that the diet of YOY perch was dominated by zooplankton and benthic macroinvertebrates for all sampling dates. In 2014, the most common prey item in fish caught at the southern shore were chironomid larvae (85.6% of investigated stomachs), followed by *D. villosus* (56.7%). In the same year, more than 70% of the fish from the sampling site “North” contained zooplankton and chironomid larvae in their stomachs, followed by *D. villosus* (61.8%). In 2016, zooplankton (97.5%), chironomid larvae (96.6%) and *D. villosus* (84.9%) were the most frequent prey items. Chironomid and culicid pupae were present in less than 50% of fish stomachs at all sampling dates. Adult stages of Ephemeroptera, Odonata and Trichoptera were rarely consumed and found in less than 5% of the stomachs. Other benthic macroinvertebrates such as snails and leeches were only present in the diet of YOY perch from the northern sampling site (Table 2). Information on the number of consumed prey items can be read from Fig. 3.

**Table 2:** Frequency of occurrence (%) of prey categories in the diet of YOY perch (*Perca fluviatilis*) from two sampling sites of Lake Müggelsee (“South” and “North”) in 2014 and 2016.

Prey categories	Frequency of occurrence (%)		
	South 2014	North 2014	North 2016
Zooplankton	45.6	70.3	97.5
*Dikerogammarus villosus*	56.7	61.8	84.9
*Chelicorophium curvispinum*	27.8	40.9	22.7
Chironomidae larvae	85.6	76.8	96.6
PS insect larvae	42.2	32.4	43.7
Pelagic macroinvertebrates	41.1	49.4	37
Meiobenthos	NP	12	78.2
Other benthic macroinvertebrates	NP	18.1	10.9
Terrestrial prey types	4.4	2.3	NP

Zoopankton: cladocerans and copepods; pelagic macroinvertebrates: Chiromonidae and Culicidae pupae; predator-sensitive (PS) insect larvae: Ephemeroptera and Plecoptera; meiobenthos: Ostracoda and Harpacticoids; other benthic macroinvertebrates: snails and leeches; terrestrial prey types: adult stages of Ephemeroptera, Odonata and Trichoptera.

*NP* not present.

In 2014, eye fluke infection intensity (corrected for fish size) was negatively correlated with the amount of zooplankton (Spearman’s rank correlation: *r*_*s*_ =  − 0.208, *n* = 90, *P* = 0.05; Fig. 3E) and positively correlated with the amount of predator-sensitive insect larvae (Spearman’s rank correlation: *r*_*s*_ = 0.216, *n* = 90, *P* = 0.041; Fig. 3I) consumed by YOY perch from the southern shore. No significant relationship was detected between eye fluke infection intensity and the consumed amount of the two amphipod species or other insect larvae (*P* > 0.05; Fig. 3A,C,G,K). In the same year at the northern shore, as eye fluke infection intensity increased, fish consumed significantly more *D. villosus* (Spearman’s rank correlation: *r*_*s*_ = 0.309, *n* = 259, *P* < 0.0001; Fig. 3B) and less *C. curvispinum* (Spearman’s rank correlation: *r*_*s*_ =  − 0.215, *n* = 259, *P* < 0.001; Fig. 3D). Infection intensity was also negatively correlated with the amount of consumed zooplankton (Spearman’s rank correlation: *r*_*s*_ =  − 0.179, *n* = 259, *P* < 0.01; Fig. 3F), chironomid larvae (Spearman’s rank correlation: *r*_*s*_ =  − 0.188, *n* = 259, *P* < 0.01; Fig. 3H) and pelagic macroinvertebrates (Spearman’s rank correlation: *r*_*s*_ =  − 0.19, *n* = 259, *P* < 0.01; Fig. 3L). No significant relationship was detected between eye fluke infection intensity and the consumed amount of predator-sensitive (PS) insect larvae (*P* > 0.05; Fig. 3J). In 2016, no significant correlation was found between infection intensity (corrected for fish size) and the amount of consumed items of any prey category (*P* > 0.05).

### Stable isotope analysis

**Figure 4:**
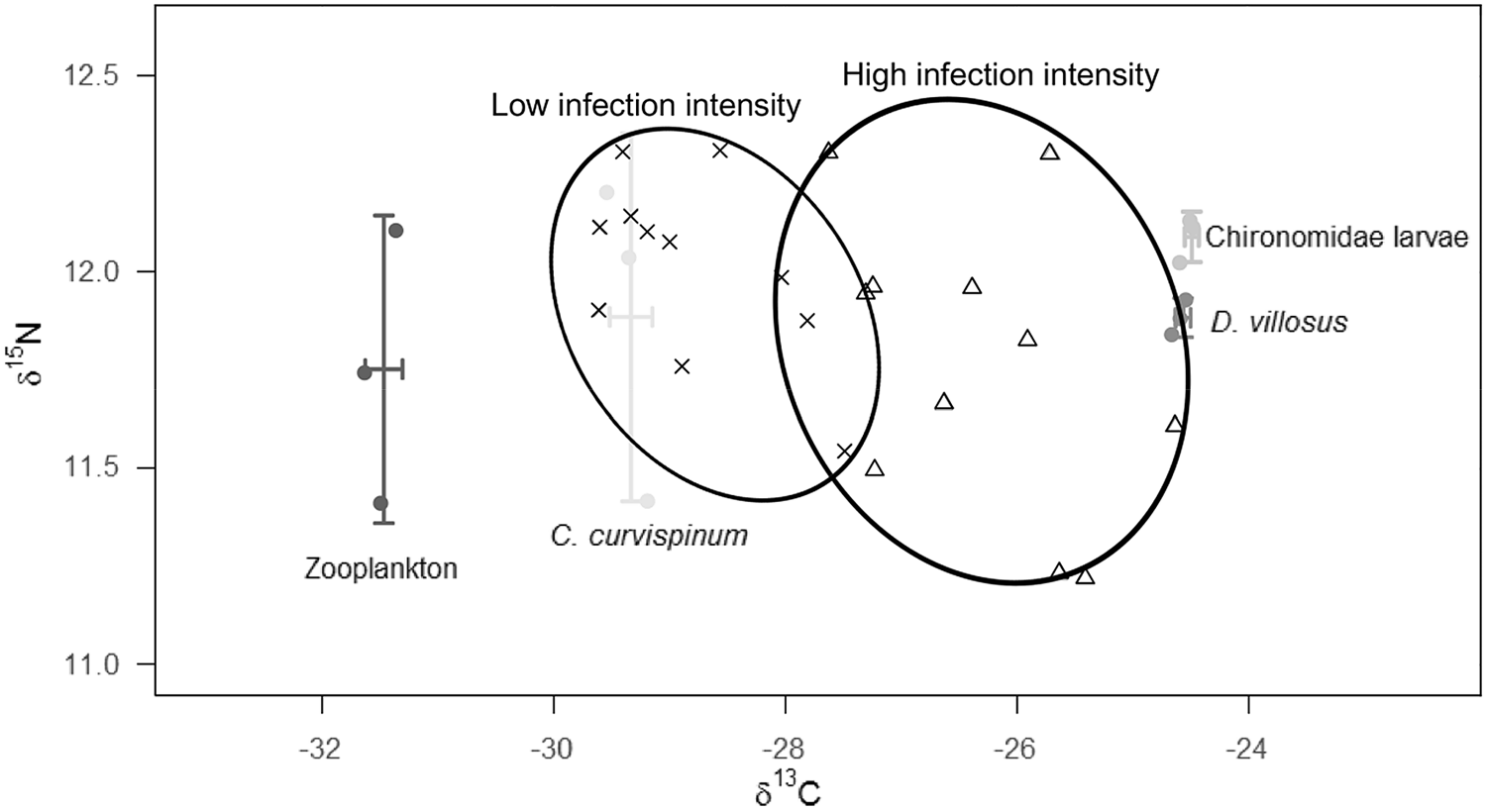
Stable isotope biplot of individual YOY perch (*Perca fluviatilis*) and their major prey categories sampled at the northern shore of Lake Müggelsee in 2016. Fish are divided in two categories based on eye fluke infection intensity: low infection intensity ( ×) = 5 ± 3 metacercariae per fish (average ± SD) and high infection intensity fish (Δ) = 39 ± 13 metacercariae per fish (average ± SD). Perch δ^13^C and δ^15^N signatures were corrected for fractionation by 1 ‰ and 3.5 ‰, respectively. Prey stable isotope signatures are shown as mean ± SD.

The results from the stable isotope analysis revealed a significant difference in the δ^13^C signatures between low and highly infected fish (*F*_1,20_ = 45.87; *P* < 0.001; Fig. 4). Less infected perch exhibited lower δ^13^C values (mean ± SD: − 28.81 ± 0.74 ‰) in comparison with more intensively infected conspecifics (− 26.34 ± 0.96 ‰). Regarding the δ^15^N signatures no significant difference between fish with low (12.01 ± 0.23 ‰) and high (11.77 ± 0.37‰) infection intensity (*F*_3,8_ = 3.24; *P* = 0.087) was observed.

Among the different prey significant differences were found for δ^13^C (*F*_3,8_ = 2789; *P* < 0.001) but not for δ^15^N (*F*_3,8_ = 0.78; *P* > 0.05). Zooplankton showed the lowest δ^13^C signature of − 31.47 ± 0.14 ‰ followed by *C. curvispinum* with − 29.33 ± 0.16 ‰. At the opposite end of the spectrum, *D. villosus* and chironomid larvae had similar δ^13^C signatures (Tukey’s test: *P* > 0.05) with the highest δ^13^C values (− 24.57 ± 0.06 ‰ and − 24.49 ± 0.05 ‰, respectively).

**Figure 5:**
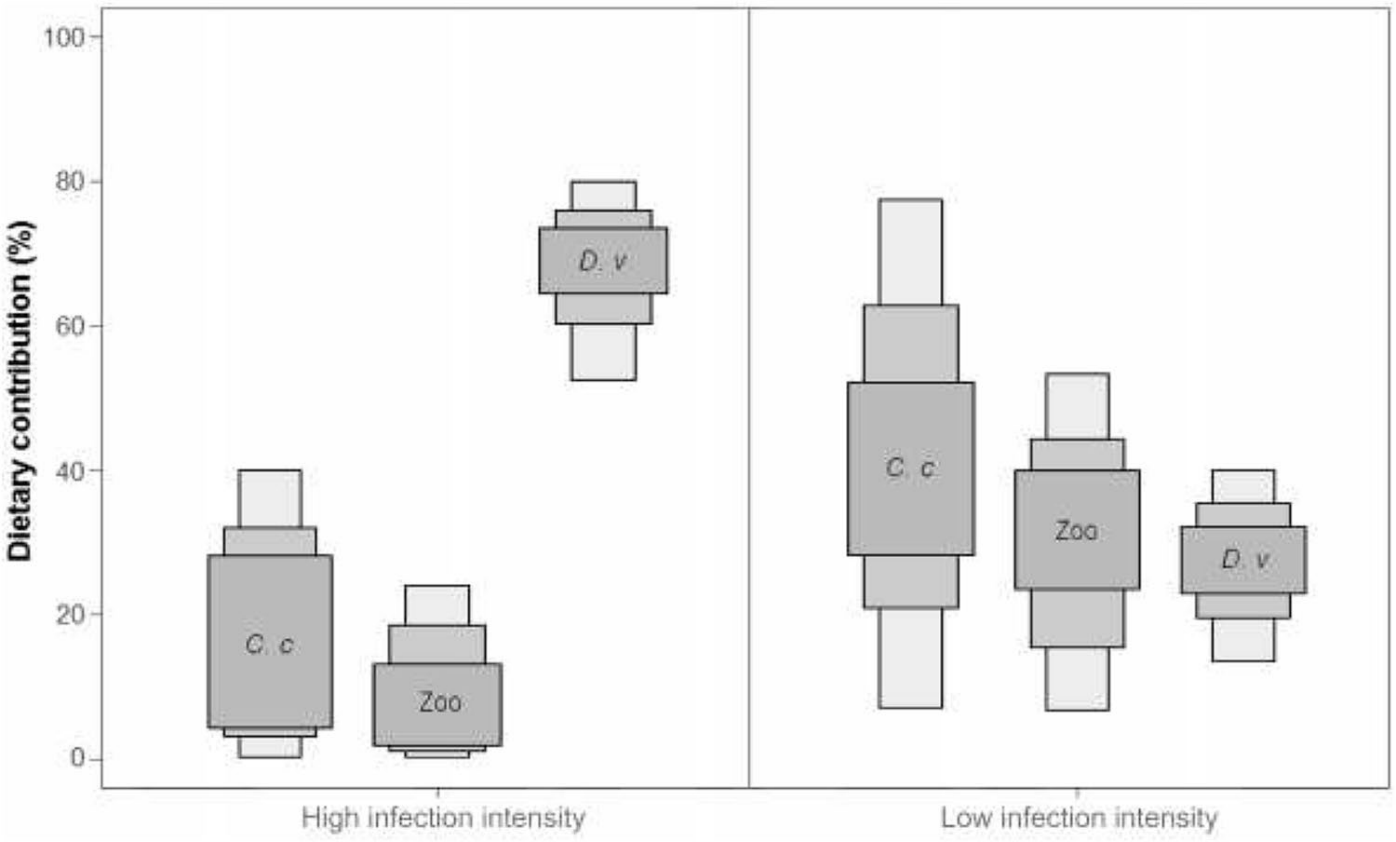
Result of SIAR Bayesian mixing model, based on δ^13^C and δ^15^N signatures, showing estimated contributions (%) of *Chelicorophium curvispinum* (C.c), zooplankton (Zoo) and *Dikerogammarus villosus* (D.v) to the diet of YOY perch (*Perca fluviatilis*) with different eye fluke infection intensity at the northern shore of Lake Müggelsee in 2016. Low infection intensity = 5 ± 3 metacercariae per fish (average ± SD) and high infection intensity = 39 ± 13 metacercariae per fish (average ± SD). The plot shows 25% (inner box), 75% and 95% (outer boxes) credibility intervals.

The Bayesian isotopic mixing model (SIAR) showed that the contribution of the prey groups to the diet of YOY perch strongly varied between individuals with high and low infection intensity. The amphipod *D. villosus* formed the bulk of highly infected perch diet (*ca.* 70%), while the dietary contributions of both *C. curvispinum* and zooplankton were considerably lower (< 20%; Fig. 5). On the other hand, low infected perch were less selective and the contributions of *D. villosus*, zooplankton and *C. curvispinum* were 27%, 35% and 38%, respectively (Fig. 5).

## Discussion

Intraspecific diet specialization is common in natural populations and may have important ecological and evolutionary consequences^54,59^. Individual diet specializations observed in European perch have been associated with variation in morphology between individuals using different habitats (littoral/pelagic)^35–38^. However, less is known about the factors that drive within-habitat differences in feeding behaviour. In general, differences in resources utilization have been considered to be a strategy to reduce intraspecific competition^39,41,54^. In the present study, both SCA and SIA provide evidence that infection with eye flukes influences prey composition in perch. Obviously, this can be attributed to the impaired visual capability, which affects prey detection ability. Eye fluke infection intensity might also affect foraging behaviour of perch within a shoal. A modified foraging behaviour eventually may represent a compensatory mechanism for reduced foraging competitive ability caused by eye fluke infection^20,23,24,60^.

The parasitological survey showed that, even though six trematode species infected the eyes of perch in Lake Müggelsee, *T. clavata* was by far the most abundant and prevalent species, infecting all but one fish examined (prevalence > 99%). Similar patterns of eye fluke infection have been previously recorded in the same lake^34^.This previous study showed that mean infection intensity of *T. clavata* in perch differed greatly between similar habitats at different locations, as it was also observed in 2014 for the sampling sites “North” and “South”. Such differences in infection intensity have been partly explained by the snail host abundance; however, other factors, such as nesting sites of the final bird host (grebes) could also contribute to the heterogeneous pattern observed in the lake^34^. Repeated samplings over time and space suggest the presence of a stable eye fluke component community dominated by *T. clavata*. Throughout Europe comparable eye fluke component communities in perch have been recorded^28,30,32,33^. In this study, potential effects of eye flukes on their host were analyzed with the whole component community considering that each species can impair the visual performance of fish. For instance, *Tylodelphys* spp. can occlude the visual field by retinal obstruction^16^ while *Diplostomum* spp. targeting the retina can damage the choroid layer, pigment epithelium and photoreceptors^61,62^. In either way, both genera of eye flukes would clearly affect visual capabilities of their fish hosts.

Parasites are often expected to reduce fish body condition due to pathological effects of infection^63,64^. The detrimental effect on the visual performance together with the reduction of feeding efficiency^15,16,20,23,24,65^ induced by eye flukes might affect fish body condition. In this study, a positive relationship between condition factor and infection intensity was observed for fish sampled in 2014 but this relationship was not detected in 2016. Whether eye flukes may impact fish condition is uncertain as the available information is contradictory. Previous studies reported negative, positive and no relationships between the condition factor of fish and infection of eye flukes^66–69^. It has been shown that the condition factor of fish varies with factors such as sex, seasonal fluctuations, spawning cycles, fullness of the stomach and even total parasite biomass^70,71^. Therefore, future research evaluating the impact of eye flukes on the condition factor of fish requires a more detailed assessment, taking also into account the other variables mentioned above in order to avoid bias, masking of actual infection effects or spurious correlations.

During ontogenetic development, perch undergo habitat and dietary shifts. After hatching, larvae move out to the pelagic zone where they feed on zooplankton. Afterwards, juvenile perch migrate to the littoral zone where they gradually change to a diet of different macroinvertebrates^42,72–76^. During this stage, diet composition of juvenile perch varies between lakes depending on habitat, food availability and the degree of inter- and intraspecific competition^35,42,43,77^. For instance, in some locations perch in the littoral feed mainly on zooplankton up to the length of ca. 15 cm (e.g.^77^) while in other locations fish completed the shift from zooplankton to benthic macroinvertebrates at smaller sizes (> 7 cm) (e.g.^78,79^). In the present study, the stomach content analysis showed that the main benthic prey items of YOY perch were amphipods and chironomids and as planktonic prey fish consumed daphnids and copepods. Presumably fish are still transitioning as both benthic and planktonic prey are important, which is in line with a previous study from Lake Müggelsee where even 1 + perch fed still on a balanced mix of both benthic and planktonic prey^50^.

Individual diet specialization among YOY perch in the littoral has been previously detected through substantial differences in carbon isotope signals^39^ and diet characterization based on stomach contents, where it was observed that some individuals feed primarily on benthic macroinvertebrates, whereas others feed mostly on zooplankton^41^. The results from SCA revealed that as eye fluke infection intensity increased the consumption of *D. villosus* and PS insect larvae (Ephemeroptera and Plecoptera) increased while the consumption of *C. curvispinum*, zooplankton and chironomids decreased. The variability in the δ^13^C-signatures observed among juvenile perch indicates the presence of individual diet preferences^53,54,80^, and in our study they were related to eye fluke infection intensity. Additionally, the results from an isotopic mixing model further support the observations from the SCA showing that more intensively infected fish had a more selective diet with a high proportion of *D. villosus*, in contrast to less intensively infected conspecifics.

It is conceivable that by impairing the visual performance eye flukes could affect prey detection, especially of small size items favoring the consumption of larger invertebrates. This effect should be intensified with infection intensity as the impact of eye flukes on fish’s visual ability is intensity-dependent^20,24,60^. A parasite-induced change in prey preferences of fish has also been observed in another parasite-host system. Three-spined stickleback females infected with *Schistocephalus solidus* fed mostly on benthic invertebrates while non-infected females of the same population fed on planktonic cladocerans^81^. *S. solidus* does not infect a sensory organ, but it decreases the foraging competitive ability by impairing swimming performance^82,83^. Thus, parasites that impair foraging competitive ability can indirectly influence prey choice of the host, which in turn may alter not only predator–prey interactions but also the host’s functional role in energy transfer through the ecosystem.

SIA did not reveal the typical diet specialization (planktivorous *vs.* benthivorous) in YOY perch. Instead less heavily infected fish seemed to be “generalists”, as the different prey categories similarly contributed to their diet, while individuals with higher infection intensity consumed more of the large *D. villosus*. Presumably, this preference may not only be driven by prey size but also by prey behaviour. Although the amphipod *C. curvispinum* is a relatively large macroinvertebrate, its importance on the diet of more intensively infected fish was low (diet contribution 17%). *C. curvispinum* is a filter-feeder, which builds mud tubes on hard substrates, such as stones, wood structures and aquatic vegetation that can provide shelter against predators^84,85^. On the other hand, *D. villosus* is an opportunistic species, well known for its predatory behaviour on a wide range of other invertebrate species^86,87^. Both laboratory and field studies have described a practically continuous feeding activity of *D. villosus* without any distinctive diurnal rhythm or extended feeding interruptions^87–89^. Accordingly, given the difference on behaviour, *D. villosus* individuals may be more conspicuous than *C. curvispinum* and presumably easier to detect for heavily infected fish because they do not hide inside mud tubes.

Chironomid larvae belong to different feeding guilds for the larvae, such as filter-feeders, grazers, detritivores and predators^90,91^ and thus ingest carbon from notably different sources such as detritus, periphyton and prey with presumably different isotopic signatures^52,92^. However, the results in the present study revealed only a low variance in isotopic signatures of chironomids. Probably, the sampling method may have led to an underrepresentation of the different feeding guilds as they were hand-picked from aquatic vegetation and stones. It is possible that the sample consisted of individuals belonging to the subfamily Tanypodinae, which have a predatory feeding behaviour and move freely on aquatic vegetation or substratum surface^90,93^. Because the uncertain composition of the chironomid sample, chironomids were excluded from the mixing model analysis and the estimation of its contribution to the diet of YOY perch was only possible on the basis of the stomach contents. The results from SCA showed a negative relationship between eye fluke infection intensity and consumption of chironomids. However, further research taking into consideration the different feeding guilds of chironomid larvae and having a more differentiated collection for stable isotopes analysis is required to unveil the role of eye fluke infection on the consumption of chironomid larvae, which are an important prey for YOY perch.

This study provides for the first time evidence that eye fluke infection intensity can significantly influence diet composition of YOY perch. Such hidden effects of parasites, leading to diet specialization among individuals within a population (dietary clusters) are underappreciated modulators in food webs^94^. Nonetheless, they may modulate a substantial amount of energy flow through the system. Therefore, in aquatic ecosystems eye fluke infection may play an important role not only in the energy transfer to upper trophic levels by increasing susceptibility to predation^21,22,95–97^, but also in the interaction of the host with lower trophic levels by altering diet composition. They may also lower top-down effects of YOY perch on zooplankton with cascading effects on phytoplankton and thus water clarity in lakes^98^. This study gathered data only for one lake; however, infection and community dynamics vary between water bodies and further research is needed to gain a generalized understanding of the complex relationships between eye flukes, hosts, communities and ecosystems. However, considering the cosmopolitan distribution of eye flukes and the diversity of fish hosts, it can be predicted that eye flukes are important players in aquatic food webs worldwide.

## Methods

### Fish sampling and examination

YOY perch were sampled in 2014 and 2016 from temperate, eutrophic Lake Müggelsee (Berlin, Germany). The lake has a disc-like shape with a relatively regular shoreline, which is characterized by the presence of dense belts of common reed (*Phragmites australis*)^99^. The fish community of this lake mainly consists of perch, roach (*Rutilus rutilus*), bream (*Abramis brama*), ruffe (*Gymnocephalus cernuus*), bleak (*Alburnus alburnus*) and pikeperch (*Sander lucioperca*)^50^. Fish were caught by electrofishing in the littoral area at two sampling sites, one located at the southern shoreline (“South”, N 52° 25′ 39′′ E 13° 38′ 14′′) and the other at the northern shoreline (“North”, N 52° 26′ 51′′ E 13° 39′ 15′′). Both sampling sites were located at the edge of extended reed belts. In 2014 fish were caught at the “South” on the 19th of August 2014 and at the “North” on the 28th of August and 9th of September. In 2016 fish were sampled only at the “North” on the 8th of August.

Immediately after capture, YOY perch were killed and placed on ice. In the laboratory, all fish were measured to the nearest 1 mm (total length, TL) and weighted to the nearest 0.01 g (wet weight, WW). The eyes were removed, dissected, and entirely examined for the presence of parasites using a stereo microscope (8×–20 × magnification). All parasites were counted and identified to the lowest taxonomic level possible based on morphological characteristics^100–104^. Specimens of the *D. baeri* complex^105^ are reported as *D. baeri* (sensu lato). Only trematode species were found and the data from both eyes was combined for each fish as metacercariae do not exhibit a preference for either left or right eye^30,33^. During the 2016 sampling, perch were additionally examined for the presence of other macro-endoparasites in the liver, digestive tract and body cavity. Prevalence and mean intensity of the parasites were calculated according to Bush et al.^106^.

Considering that fish caught at the “North” in the two sampling dates of 2014 had similar size range and parasite loads, these two samples were combined. Morphological parameters of the sampled fish are summarized in Table 3. Fulton’s condition factor (*K*) for each fish was calculated according to the Eq. (1) after Nash et al.^107^

1 $$K = \, 100 \, \times \, \left( {{\text{W }}/{\text{ TL}}^{3} } \right)$$

where W = wet weight (g) and TL = total length (cm).

**Table 3:** Number of young-of-the-year (YOY) perch (*Perca fluviatilis*) sampled from Lake Müggelsee (Berlin, Germany) with their respective sampling year and site.

Sampling year	Sampling site	*n*	TL (cm) Mean ± SD	WW (g) Mean ± SD
2014	“South”	90	6.0 ± 0.5	2.1 ± 0.6
2014	“North”	259	6.2 ± 0.8	2.2 ± 1.0
2016	“North”	119	5.1 ± 0.5	1.4 ± 0.4

Total length (TL) and wet weight (WW) are given as means ± SD.

### Stomach content analysis

The stomachs were removed and preserved in 70% ethanol. Stomach contents of each fish were identified to order, family, or species and counted under a stereo microscope (8×–20× magnification). Most of the prey items were intact and easy to determine and count. However, if prey items were broken, for instance chironomid larvae, only the heads were counted to quantify the prey number, as this body part is easily detectable.

Prey items were separated into nine categories: (1) zooplankton (cladocerans and copepods), (2) *Dikerogammarus villosus*, (3) *Chelicorophium curvispinum*, benthic insect larvae were separated into two groups: (4) Predator-sensitive (PS) insect larvae, which consisted of organisms living on macrophytes, branches or on other substrates and included Ephemeroptera and Plecoptera. These taxa are relatively large, conspicuously visible and thus sensitive to fish predation^108^; the other group was (5) Chironomid larvae, which are often cryptically coloured, tube-builders living on or within the substrate^109^, making them less sensitive to visual fish predation^108^. Further prey categories were (6) pelagic macroinvertebrates (pupae of Chiromonidae and Culicidae), (7) Meiobenthos, (8) other benthic macroinvertebrates (snails and leeches) and (9) terrestrial prey types (adult stages of Ephemeroptera, Odonata and Trichoptera).

### Stable isotope analysis

In 2016 samples of muscle tissue were taken from under the dorsal fin of each fish and frozen at –20 °C for SIA. A total of 22 fish were selected to evaluate their isotopic signatures (δ^13^C and δ^15^N). Individuals were chosen according to the eye fluke infection intensity and divided into two groups. Eleven fish with the lowest infection intensity formed the low infected group (mean intensity ± SD: 5 ± 3 metacercariae per fish). Further 11 fish of comparable size (TL), but harboring high numbers of eye flukes were chosen to form the high infected group (mean intensity ± SD: 39 ± 13 metacercariae per fish). TL was also considered for the selection as stable isotope ratios increase with body length in perch^79,110^ and to exclude a size effect that might reflect the ontogenetic diet shift. TL of the selected fish ranged from 4.0 to 4.9 cm and there was no size difference between the two groups (Mann–Whitney U-test: W = 45.5, *n *_*1*_ = *n *_*2*_ = 11, *P* = 0.33). Among the selected fish, macro-endoparasite species exhibited low prevalence with the exception of *Ichthyocotylurus* sp. (see Supplementary Table S1). However, infection intensity of *Ichthyocotylurus* sp. did not differ between the two groups (Mann–Whitney U-test: W = 78.5, *n*_*1*_ = *n*_*2*_ = 11, *P* = 0.25).

Additionally, zooplankton and benthic macroinvertebrates were collected in August 2016 from the sampling site “North” to estimate prey isotopic signatures. Zooplankton was caught using a conical plankton net (100 μm mesh) and, considering the data from SCA, a mixed sample consisting approximately of two thirds of daphnids and one third of copepods was prepared. Benthic invertebrates, *D. villosus*, *C. curvispinum* and chironomid larvae, were hand-picked from aquatic vegetation and stones. After collection prey samples were frozen at –20 °C.

Prior to the analysis, all samples were freeze-dried and grounded to a fine powder. Then, per sample triplicates of 400–700 μg were weighed into 4 × 6 mm tin foil capsules for solids (IVA Analysentechnik e.K., Meerbusch, Germany). The samples were analyzed using an elemental analyser (PYRO Cube EA; Elementar Analysensysteme, Langenselbold, Germany) coupled with an isotope ratio mass spectrometer (IsoPrime 100 IRMS; Elementar Analysensysteme Langenselbold, Germany). The measurements were carried out as described by Nachev et al.^111^ and the results were obtained following the principle of identical treatment and normalization according to Werner and Brand^112^.

Isotope ratios are expressed in the δ-notation, in per mil units (‰), which describes the isotope ratio in the sample in relation to an international reference substance, according to the Eq. (2).

2 $$\updelta ^{h} E_{s,ref} = \, \left[ {\left( {R\left( {^{h} E/^{l} E} \right)_{s} /R\left( {^{h} E/^{l} E} \right)_{ref} } \right) \, - \, 1} \right] \, \times \, 10^{3}$$

where R(^h^E/^l^E)_s_ is the ratio of the heavy and light isotope (here ^13^C/^12^C as well as ^15^ N/^14^ N) in the sample, and R(^h^E/^l^E)_ref_ is the ratio in the reference material. The normalization of the laboratory internal standard (acetanilide) was performed using international standards USGS40 and USGS41 (both International Atomic Energy Agency, Vienna). The instrument drift was controlled and corrected with the internal standard, whereas after every three replicates an acetanilide standard was measured.

### Data analysis

Sampling dates and sites were analyzed separately because it can be assumed that prey availability varies over space and time. A linear regression was used to investigate the relationship between eye fluke infection intensity and perch size (TL) per sampling date. Since significant relationships were found, to correct for fish size the residuals from the regressions were used as predictor in the evaluation of perch’s condition factor and prey composition. The relationships between infection intensity corrected for fish size (the residuals of the regressions) with both condition factor and the number of consumed items for each prey category (log x + 1 transformed), present at all sampling dates, were analyzed using Spearman rank correlation. Significant correlations where accepted when *P* ≤ 0.05.

For the analysis of δ^13^C- and δ^15^N-values of perch, correction for trophic fractionation by 1 ‰ for carbon and 3.5 ‰ for nitrogen was used^113–118^.

To compare the isotopic signatures among perch, linear models (LMs) were carried out including infection intensities of eye flukes, *Ichthyocotylurus* sp. and its interaction as descriptors. Because neither *Ichthyocotylurus* sp. nor the interaction term contributed significantly to the LMs of δ^13^C- and δ^15^N-values (δ^13^C: *F*_3,18_ = 14.16, *P* < 0.0001; *Ichthyocotylurus* sp. β = − 0.28, *P* = 0.154; interaction β = 0.41, *P* = 0.08. δ^15^N: *F*_3,18_ = 3.20, *P* < 0.048; *Ichthyocotylurus* sp. β = 0.077, *P* = 0.78; interaction β = − 0.56, *P* = 0.104), LMs were fitted only with eye fluke infection intensity (low *vs.* high) as independent variable. Differences in isotopic signatures between prey types were also evaluated using LMs, with prey categories (*D. villosus*, *C. curvispinum*, chironomid larvae, and zooplankton) as independent variables. Residuals were tested for normality with a Wilk-Shapiro test and qq-plots. Tukey’s post hoc test was applied to determine significant differences in the δ^13^C-values between prey categories.

To quantify the proportion of different prey items in diets of low and highly infected perch, a stable isotope mixing model was run using the package SIAR^119^ for R. SIAR calculates the most likely set of dietary proportional contributions within a Bayesian framework, based upon the isotopic ratios in a set of potential food sources and consumers^57^. SIAR was set to run one million iterations, thinned by 300 and with an initial discard (burn in) of 400,000 iterations.

Since both δ^13^C- and δ^15^N values of chironomids were not significantly different from those of *D. villosus* and because different feeding modes exist within subfamilies of chironomid larvae (e.g. detritivores, filter feeders, grazers and predators)^90,91^, while sampled chironomids used for SIA were not identified to subfamily level, SIAR results were obtained excluding this prey type. Furthermore, samples of PS insect larvae were not available for SIA and could not be included in the evaluation.

All statistical analyses were performed using R version 3.5.1^120^.

### Ethics statement

Sampling of the fish for this study was approved by the responsible fisheries authority (Fischereiamt Berlin). Fish were sacrificed by severing the spinal cord after sedation in accordance with the relevant guidelines and regulations (Directive 2010/63/EU).

## Supplementary Information

Supplementary Information.

## Data Availability

The datasets generated during the current study are available from the corresponding author on reasonable request.
