# Supplementary material for: Eye fluke infection changes diet composition in juvenile European perch (Perca fluviatilis)
Source: Sci Rep. 2021 Feb 9;11:3440. doi: 10.1038/s41598-021-81568-y (PMC7873217; doi:10.1038/s41598-021-81568-y)
Supplement: Supplementary file 1 — Supplementary Information. [file 41598_2021_81568_MOESM1_ESM.pdf]

Eye fluke infection changes diet composition in juvenile European perch (*Perca fluviatilis*)

Supplementary Information

Jenny C. Vivas Muñoz [1,2] *, *Christian K. Feld* [3,4], *Sabine Hilt* [1,2], *Alessandro Manfrin* [3,5,6], *Milen Nachev* [3,4], *Daniel Köster* [7], *Maik A. Jochmann* [4,7], *Torsten C. Schmidt* [4,7], *Bernd Sures* [3,4], *Andrea Ziková* [1], *Klaus Knopf* [1,2]

[1] Leibniz-Institute of Freshwater Ecology and Inland Fisheries, Müggelseedamm 310, 12589 Berlin, Germany

[2] Faculty of Life Sciences, Humboldt University, Invalidenstrasse 42, 10115 Berlin, Germany

[3] Aquatic Ecology, University of Duisburg-Essen, Universitätsstr. 5, 45141 Essen, Germany

[4] Centre for Water and Environmental Research, University of Duisburg-Essen, Universitätsstr. 5, 45141 Essen, Germany

[5] Environmental Campus Birkenfeld, University of Applied Sciences Trier, Post Box 1380, 55761 Birkenfeld, Germany

[6] Institute for Environmental Sciences, University of Koblenz-Landau, Fortstrasse 7, 76829 Landau/Pfalz, Germany

[7] Instrumental Analytical Chemistry, University of Duisburg-Essen, Universitätsstr. 5, 45141 Essen, Germany

E-mail addresses: vivas.munoz.je@gmail.com (J.C. Vivas Muñoz), christian.feld@uni-due.de (C.K. Feld), hilt@igb-berlin.de (S. Hilt), manfrin@uni-landau.de (A. Manfrin), milen.nachev@uni-due.de (M. Nachev), daniel.koester@uni-due.de (D. Köster), maik.jochmann@uni-due.de (M.A. Jochmann), torsten.schmidt@uni-due.de (T.C. Schmidt), bernd.sures@uni-due.de (B. Sures), zikova@igb-berlin.de (A. Ziková), klaus.knopf@igb-berlin.de (K. Knopf).

Parasite species	Number of infected fish		Infection intensity (parasites per infected fish)	
	**Low**	**High**	**Low**	**High**
Triaenophorus nodulosus	1		1	
Camallanus sp.		1		1
Proteocephalus percae		1		2
Bunodera luciopercae		3		1.3
Anguillicola crassus (L3)	1		1	
Ichthyocotylurus sp.	9	9	9	5.3

Table S1. Macro-endoparasites infecting YOY perch (*Perca fluviatilis*) sampled at the northern shore of Lake Müggelsee in 2016 and selected for the stable isotope analysis (SIA). Fish are divided in two categories (each with a total number of 11 individuals) based on eye fluke infection intensity: low infection intensity (**Low**) = 5 ± 3 metacercariae per fish (average ± SD) and high infection intensity fish (**High**) = 39 ± 13 metacercariae per fish (average ± SD).
